# Vision-Related Quality of Life among Adult Patients with Visual Impairment at University of Gondar, Northwest Ethiopia

**DOI:** 10.1155/2020/9056097

**Published:** 2020-03-23

**Authors:** Betelhem Temesgen Yibekal, Destaye Shiferaw Alemu, Dereje Hayilu Anbesse, Abiy Maru Alemayehu, Yezinash Addis Alimaw

**Affiliations:** Department of Optometry, School OF Medicine, College of Medicine and Health Science, University of Gondar, Gondar City, Ethiopia

## Abstract

**Purpose:**

The purpose of this study was to assess vision-related quality of life and associated factors among adult patients with visual impairment at the University of Gondar Tertiary Eye Care and Training Center, Northwest Ethiopia.

**Methods:**

The institution-based cross-sectional study was conducted from April 24 to May 12, 2017, at the University of Gondar Tertiary Eye Care and Training Center among 484 patients with visual impairment. Pretested, structured National Eye Institute Visual Function Questionnaire-25 was used to collect data by interviewing. Data were entered using EPI-INFO version 3.5.1 and analyzed with SPSS version 20. Binary logistic regression was used to determine factors associated with vision-related quality of life. Variables with *p* value <0.05 in multivariable logistic regression were considered as statistically significant.

**Result:**

A total of 484 study subjects participated with a response rate of 98.9%. The median age of the participants was 60 years with the interquartile range of 25 years. The proportion of poor vision-related quality of life was 238 (49.2%) (95% CI: 44.2%–53.3%). Age >75 years (AOR = 1.87 (95% CI: 1.02–3.40)), rural residency (AOR = 1.71 (95% CI: 1.13–2.60)), severe visual impairment/blindness (AOR = 2.76 (95% CI: 1.80–4.23)), and history of visual impairment longer than 3 years (AOR = 2.85 (95% CI: 1.61–5.04)) had statistically significant association with poor vision-related quality of life.

**Conclusion:**

Almost half of the patients with visual impairment had poor vision-related quality of life. Severe visual impairment/blindness, long duration of visual impairment, older age, and rural residency had a statistically significant association with poor vision-related quality of life.

## 1. Introduction

Vision-related quality of life (VRQOL) is defined as a person's satisfaction with their visual ability and how their vision impacts on their daily life [1]. Since it is a broad concept, it can be affected in a complex way by the person's physical health, psychological state, level of independence, and social relationships [2].

Vision has a vital role for best performance in functional and social life. Eyesight/vision accounts for about 80% of the function of all the five senses combined [3]. Hence, visual impairment leads to a restriction in all areas of life and, in particular, VRQOL by reducing activities associated with participation in society and religion, mobility, recreation, daily living, and intense visual tasks [4, 5]. In addition, visual impairment is associated with depression, frustration, and anxiety not only because of the impairment but also because of the accompanying worry that the condition may worsen or the difficulty in adjusting to reduced activity [6].

Visual impairment is a global public health problem which leads to a variety of public health, social, and economic problems, especially in developing countries where over 90% of the world's individuals with visual impairment live. In sub-Saharan Africa, the average prevalence of blindness is about 1.4% [7]. Based on the presenting visual acuity, the prevalence of visual impairment in Ethiopia is 5.3% [8].

Despite the higher prevalence of visual impairment in Ethiopia [8], there is scarce information on VRQOL among people with visual impairment. Evaluation of the influence of visual impairment on daily activities, emotional state, social participation, and mobility is very valuable. However, there is limited information on VRQOL and associated factors in Ethiopia in general and the study area in particular. Therefore, this study aims to determine VRQOL and associated factors among adult patients with visual impairment.

Assessment of VRQOL provides a general overview of the impact of the visual impairment on a patient's life from the patient's perspective.

## 2. Materials and Methods

### 2.1. Study Design, Setting, and Sampling

An institution-based cross-sectional study was conducted from April 24 to May 12, 2017. The study was conducted at the University of Gondar Tertiary Eye Care and Training Center. This eye care center provides a comprehensive clinical and community eye health services for eight zones and serves as a major referral center for 14 million people living in Northwest Ethiopia. It is the only tertiary eye care center for the population in Northwest Ethiopia. It has five special clinics (anterior segment, pediatric and strabismus, vitro-retina, glaucoma, and refraction).

A sample size with a total of 489 patients was determined using OpenEpi computer software with single population proportion formula considering total population of 1694 which is the total number of patients seen monthly (*p* = 50%) because there are no data on the proportion of poor quality of life among patients with visual impairment and margin of error = 4%. The generated sample size was found to be *n* = 444. Considering 10% for the nonresponse rate, the total sample size was 489.

All consecutive patients (both new and patients on follow-up) who came to the University of Gondar Tertiary Eye Care and Training Center during the study period aged 18 years and above were included in the study.

The study was conducted in accordance with the Declaration of Helsinki and approved by the University of Gondar Ethical Review Board. Ethical clearance was obtained from the University of Gondar, College of Medicine and Health Science, School of Medicine Ethical Review Committee. Informed verbal consent was obtained from each respondent. Oral informed consent was considered since the data were collected by using an interview administered structured questionnaire and also there was no invasive examination procedure conducted for the patients for the sake of this research. Patient information was obtained with no identifier and confidentiality was maintained.

### 2.2. Operational Definitions

The World Health Organization (WHO) classification of vision was used:

1. Visual impairment: presenting distance visual acuity of less than 6/18 on the better eye using a Snellen chart placed 6 meters away from the participant
2. Moderate visual impairment: presenting distance visual acuity of less than 6/18 to 6/60 on the better eye using a Snellen chart placed 6 meters away from the participant
3. Severe visual impairment: presenting distance visual acuity of less than 6/60 to 3/60
4. Blind: presenting distance visual acuity of less than 3/60 to no light perception
5. Poor vision-related quality of life: individuals who scored less than the overall mean in the National Eye Institute Visual Function Questionnaire-25 (NEI VFQ-25) score are considered to have poor vision-related quality of life
6. Good vision-related quality of life: individuals who scored the overall mean and above in the National Eye Institute Visual Function Questionnaire-25 scores are considered to have good vision-related quality of life

### 2.3. Data Collection

Data were collected using a pretested, structured questionnaire consisting of questions for sociodemographic factors; vision-related quality of life (VRQOL) of people with visual impairment, and associated factors of poor VRQOL. Face-to-face interview to estimate VRQOL and patients' medical chart review to determine clinical factors was employed by trained optometrists. The ocular conditions were determined after the patients had anterior and posterior segment examination. Then, we took the ocular disease which best explains the patients' visual reduction. For the cases which have more than one disease which can cause a visual reduction, we considered professional agreement done by three senior ophthalmologists and took the agreed cause of visual impairment which best explains patients' visual reduction as an ocular condition when two or more ophthalmologists agree. National Eye Institute Visual Functioning Questionnaire- 25 (NEI VFQ-25) which contains 25 items under 12 subscales was used to determine VRQOL.

### 2.4. Statistical Analysis

The coded data were checked, cleaned, and entered into EPI-INFO 3.5.1 and exported into SPSS version 20 for analysis. Descriptive statistics such as proportion, frequency, ratios, and summary statistics (mean, standard deviation, and range) were calculated. Binary logistic regression was performed to determine factors associated with poor VRQOL. Variables with *p* value <0.05 at multivariable regression were considered as statistically significant. Adjusted odds ratio (AOR) with 95% confidence interval was used to assess the strength of the association.

## 3. Results

A total of 484 people with visual impairment participated with a response rate of 98.9%. Among study participants, 283 (58.5%) were males. The median age of the participants was 60 years with the interquartile range of 25 years. More than half of the participants (297 (61.4%)) were rural residents (Table 1).

One hundred seventy-five (36.2%) of the participants had severe VI/blindness and 202 (41.7%) of participants had a history of VI for more than three years. Ninety-seven (20%) of the study participants had systemic comorbidities (Table 2).

**Figure 1:**
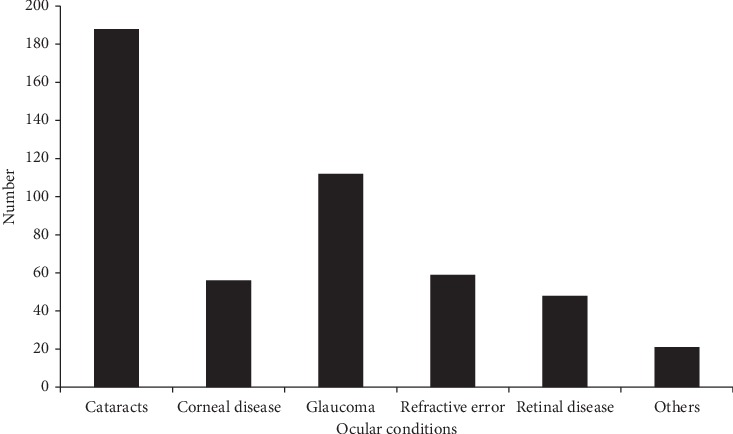
Ocular conditions of study participants at the University of Gondar Tertiary Eye Care and Teaching Center, Ethiopia, 2017 (*n* = 484). Other ocular conditions include phthisis bulbi and uveitis.

Cataract was the most common ocular condition among study participants, 188 (38.8%). (Figure 1).

**Figure 2:**
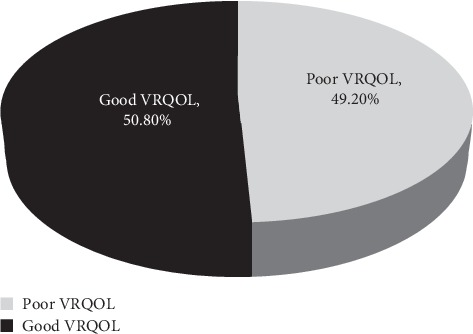
Vision-related quality of life of study participants at the University of Gondar Tertiary Eye Care and Teaching Center, Ethiopia, 2017 (*n* = 484).

In this study, 238 (49.2%) (95% CI: 44.2%–53.3%) of patients with visual impairment had poor overall vision-related quality of life (Figure 2).

Among the 12 subscales, general health (65.7%) was the most affected subscale and least affected was color vision (41.9%) (Table 3).

The results of multivariable logistic regression analysis showed that poor VRQOL was significantly associated with age, residence, level of VI, and duration of visual impairment. As a result, participants aged greater than 75 years were 1.87 times more likely to have poor VRQOL compared to those who were less than 45 years old (AOR = 1.87 (95% CI: 1.02–3.40)). Study participants from rural areas were 1.71 times more likely to have poor VRQOL compared with urban resident participants (AOR = 1.71 (95% CI: 1.13–2.60)). This study also shows that patients with severe visual impairment/blindness were 2.76 times more likely to have poor VRQOL compared to those who had moderate visual impairment (AOR = 2.76 (95% CI: 1.80–4.23)). Moreover, participants with a history of visual impairment for more than 3 years were 2.85 times more likely to have poor VRQOL compared to those who had a history of visual impairment for less than 1 year (AOR = 2.85 (95% CI: 1.61–5.04)) (Table 4).

## 4. Discussion

Overall the proportion of poor VRQOL in this study was 49.2% (95% CI: 44.2%–53.3%). This result is higher than that in studies conducted in Ibadan, Nigeria (21.5%) [3]. This might be due to the small sample size of patients with visual impairment. Additional reasons for this variation might be differences in lifestyles, economic status, health care system, and cultural value.

However, the result of this study is lower than that in other studies conducted in Philadelphia, USA, and Kenya [9, 10]. This might be due to the instrument they used to assess VRQOL which was time trade-off utility measure. Time trade-off provides a measure of the VRQOL associated with a health state. That is, the patients were asked the maximum number of years they would be willing to give up if they could have normal vision in both eyes for the remainder of their life. However, the instrument used in this study measures mainly VRQOL.

In this study, patients with severe visual impairment/blindness were 2.76 (AOR = 2.76 (95% CI: 1.80–4.23)) times more likely to have poor VRQOL compared to those who had moderate visual impairment. This result agrees with other studies [11–13]. This might be due to that as the visual acuity decreases, daily activities might be compromised thereby affecting social and economic status, increasing dependency and poor emotional wellbeing leading to have poor VRQOL [14].

The result of this study also showed that participants aged greater than 75 years were 1.87 (AOR = 1.87 (95% CI: 1.02–3.40)) times more likely to have poor VRQOL compared to those who were less than 45 years old. This might result from most older people considering that visual loss is to be expected in later life and think that nothing can be done to improve the situation. This result is consistent with other studies [3, 15, 16]. This might be due to that various age-related ocular disorders result in a decline in visual function, and thus have significant effects on patients' VRQOL [17, 18].

The present study found that rural residents were 1.71 (AOR = 1.71 (95% CI: 1.13–2.60)) times more likely to have poor VRQOL compared to urban residents. This result agrees with that of studies conducted in Nigeria [13] and Timor-Leste [16]. This might be due to patients who live in rural areas may not seek medical attention for eye problems for early detection and treatment, seeking care very late after the disease gets severe [19, 20]. Moreover, rural patients may have less monthly income, for example, in this study, 30% of rural patients have less than 400 ETB monthly income.

Study participants with a history of visual impairment for more than 3 years were 2.85 (AOR = 2.85 (95% CI: 1.61–5.04)) times more likely to have poor VRQOL compared to those who had visual impairment for less than 1 year. This finding is in agreement with a study conducted in Kenya [10] but different from the result of a study in Philadelphia, USA [21]. The discrepancy observed here might be due to that visually impaired persons in Philadelphia may have sufficient resources to adapt to their visual impairment over time using low vision devices, thus reducing the impact of visual impairment on VRQOL [22].

Overall this study implicates that greater efforts should be exerted to reinforce early preventative and rehabilitative programs to prevent longitudinal deterioration in vision loss which sequentially can cause poor VRQOL.

This study has some limitations. Firstly, since the study design was cross-sectional, there was lack of assessment of VRQOL over time. Secondly, the visual impairment classification in this study was based on only presenting distance visual acuity and other aspects of visual functioning (e.g., contrast sensitivity, visual field, color vision, and stereoacuity) were not assessed and may lead to inconsistent results with other study findings, and thirdly, duration of visual impairment was based on self-report and exposed to recall bias. Lastly, the study did not assess qualitative information from the patients. Almost half of the patients with visual impairment had poor VRQOL. Severe visual impairment/blindness, long duration of visual impairment, older age, and rural residency had a statistically significant association with poor VRQOL.

## 5. Conclusion

Almost half of the patients with visual impairment had poor vision-related quality of life. Severe visual impairment/blindness, long duration of visual impairment, older age, and rural residency had a statistically significant association with poor vision-related quality of life.

## Figures and Tables

**Table 1:** Sociodemographic characteristics of study participants at the University of Gondar Tertiary Eye Care and Training Center, Ethiopia, 2017 (*n* = 484).

Variables	Frequency	Percentage
Age (years)		
18–45	119	24.59
45–60	120	24.79
60–75	146	30.17
75–96	99	20.45

Sex		
Male	283	58.47
Female	201	41.53

Residence		
Rural	297	61.36
Urban	187	38.64

Educational level		
Unable to read and write	275	56.82
Only able to read and write	93	19.21
Primary education	56	11.57
Secondary education	27	5.58
College/university	33	6.82

Marital status		
Married	330	68.18
Single	54	11.16
Divorced	36	7.44
Widowed	64	13.22

Religion		
Christian	449	92.77
Muslim	35	7.23

Occupation		
Farmer	196	40.50
Student	32	6.61
Housewife	142	29.34
Merchant	33	6.81
Daily labor	32	6.61
Government employee	29	6.00
Retired	20	4.13

Monthly income		
<400	139	28.72
400–600	104	21.49
600–1500	120	24.79
>1500	121	25.00

Ethnicity		
Amhara	420	86.78
Tigrie	30	6.20
Kimant	34	7.02

**Table 2:** Clinical characteristics of study participants at the University of Gondar Tertiary Eye Care and Teaching Center, Ethiopia, 2017 (*n* = 484).

Variables	Frequency	Percentage
Visual impairment		
Moderate	309	63.84
Severe/blind	175	36.16

Duration of visual impairment		
<1 year	89	18.39
1–3 years	193	39.88
>3 years	202	41.73

Systemic comorbidities		
No	387	79.96
Yes	97	20.04

**Table 3:** NEI VFQ-25 scores across subscales among study participants at the University of Gondar Tertiary Eye Care and Training Center, Ethiopia, 2017 (*n* = 484).

Variables	Mean ± SD	Poor VRQOL frequency (%)	Good VRQOL frequency (%)
General health	(50.46 ± 30.83)	318 (65.7)	166 (34.3)
General vision	(34.74 ± 20.88)	241 (49.8)	243 (50.2)
Ocular pain	(67.08 ± 29.01)	227 (46.9)	257 (53.1)
Near activities	(53.17 ± 32.58)	273 (56.4)	211 (43.6)
Distance activities	(52.56 ± 24.87)	266 (53.7)	224 (46.3)
Social functioning	(69.44 ± 31.73)	211 (43.6)	273 (56.4)
Mental health	(55.10 ± 30.78)	222 (45.9)	262 (54.1)
Role difficulties	(43.35 ± 37.50)	260 (53.7)	224 (46.3)
Dependency	(57.88 ± 33.84)	211 (43.6)	273 (56.4)
Color vision	(75.57 ± 33.17)	203 (41.9)	281 (58.1)
Peripheral vision	(59.21 ± 34.66)	254 (52.5)	230 (47.5)
Overall VRQOL	(56.90 ± 22.18)	238 (49.2)	246 (50.8)

**Table 4:** Factors associated with poor vision-related quality of life among study participants at the University of Gondar Tertiary Eye Care and Training Center, Ethiopia, 2017 (*n* = 484).

Variable	Vision-related quality of life		COR (95% CI)	AOR (95% CI)
	Poor	Good		
Age (years)				
18–45	51	68	1.00	1.00
45–60	42	78	0.72 (0.43–1.21)	0.71 (0.40–1.24)
60–75	80	66	1.62 (0.99–2.63)	1.19 (0.70–2.04)
75–96	65	34	2.55 (1.47–4.42)	1.87 (1.02–3.40)^*∗*^

Sex				
Male	143	140	1.00	
Female	95	106	0.88 (0.61–1.26)	

Residence				
Rural	163	134	1.82 (1.25–2.63)	1.71 (1.13–2.60)^*∗*^
Urban	75	112	1.00	1.00

Educational level				
Unable to read and write	149	126	1.61 (0.77–3.33)	
Only able to read and write	44	49	1.22 (0.55–2.72)	
Primary education	19	37	0.70 (0.29–1.69)	
Secondary education	12	15	1.09 (0.39–3.03)	
College/university	14	19	1.00	

Marital status				
Married	170	160	1.00	
Single	25	29	0.81 (0.46–1.45)	
Divorced	16	20	0.75 (0.38–1.50)	
Widowed	27	37	0.69 (0.40–1.18)	

Occupation				
Farmer	108	88	1.00	
Student	10	22	0.37 (0.17–0.82)	
Housewife	75	67	0.91 (0.59–1.41)	
Merchant	9	24	0.31 (0.14–0.69)	
Daily labor	18	14	1.05 (0.49–2.22)	
Government employee	11	18	0.50 (0.22–1.11)	
Retired	7	13	0.44 (0.17–1.15)	

Monthly income				
<400	82	57	2.27 (1.38–3.73)	
400–600	49	55	1.40 (0.83–2.39)	
600–1500	60	60	1.57 (0.94–2.63)	
>1500	47	74	1.00	

Visual impairment				
Moderate	114	195	1.00	1.00
Severe/blind	124	51	4.16 (2.79–6.20)	2.76 (1.80–4.23)^*∗∗*^

Duration of visual impairment				
<1 year	31	58	1.00	1.00
1–3 years	73	120	1.14 (0.67–1.92)	1.02 (0.58–1.78)
>3 years	134	68	3.69 (2.18–6.23)	2.85 (1.61–5.04)^*∗∗*^

Ocular conditions				
Cataract	108	80	1.00	
Corneal disease	30	26	0.86 (0.47–1.56)	
Glaucoma	53	59	0.67 (0.42–1.07)	
Refractive error	19	40	0.35 (0.19–0.65)	
Retinal disease	20	28	0.53 (0.28–1.15)	
Others	8	13	0.46 (0.18–1.15)	

Systemic comorbidities				
No	190	197	1.00	
Yes	48	49	1.02 (0.65–1.59)	

^*∗*^ *p* value <0.05; ^*∗∗*^*p* value <0.001.

## Data Availability

The data sets generated and analyzed for the current study are available from the corresponding author upon reasonable request.

## Supplementary Materials

Supplementary Materials This section contains the instrument used for data collection purpose. It has both the English version and the Amharic (local language in the study area) version.
