# Supplementary material for: Vision-Related Quality of Life among Adult Patients with Visual Impairment at University of Gondar, Northwest Ethiopia
Source: J Ophthalmol. 2020 Mar 23;2020:9056097. doi: 10.1155/2020/9056097 (PMC7125459; doi:10.1155/2020/9056097)
Supplement: Supplementary Materials — This section contains the instrument used for data collection purpose. It has both the English version and the Amharic (local language in the study area) version. [file 9056097.f1.docx]

**Part: I**

**Socio-demographic characteristics**

| S.no | Questions | Response |
| --- | --- | --- |
| 101 | Age in year | ------------------------------ |
| 102 | Sex | 1. Male 2. Female |
| 103 | Residence | 1. Urban 2. Rural |
| 104 | Marital status | 1. Single 2. Married 3. Divorced 4. Widowed |
| 105 | Ethnicity | 1. Amhara  2.Oromo  3. Tigre  4. Others (specify)------------ |
| 106 | Religion | 1. Christian 2. Muslim |
| 107 | Educational status | 1.unable to read and write  2. Read and write only  3.1-8th grade  4. 9-12th grade  5. college/university |
| 108 | Occupation | 1. Student  2. House wife  3. Merchant  4. Daily labor  5. Farmer  6. Government employee  7. Retired  8.Others (specify)------------------- |
| 109 | Monthly income in birr |  |

**Part II**

**Clinical characteristics**

| 201 | Presenting distance visual | OD ___________ | OS -------------------- |
| --- | --- | --- | --- |
| 202 | Duration of VI in months | OD ___________ | OS -------------------- |
| 203 | Ocular conditions | OD1-Cataract  2-Corneal disease  3-Glaucoma  4-Refractive error  5-Retinal diseases  6- other (specify)  ----------------- | OS 1-Cataract  2-Corneal disease  3-Glaucoma  4-Refractive error  5-Retinal diseases  6-other (specify)  ---------------------- |
| 205 | Systemic comorbidities | 1. Yes 2. No | |

**Part III**

National Eye Institute Visual Functioning Questionnaire – 25

PART 1 - GENERAL HEALTH AND VISION

1. In general, would you say your overall health is:
2. Excellent
3. Very Good
4. Good
5. Fair
6. Poor
7. At the present time, would you say your eyesight using both eyes (with glasses if you wear them) is
8. Excellent
9. Good
10. Fair
11. Poor
12. Very Poor
13. Completely Blind
14. How much of the time do you worry about your eyesight?
15. None of the time
16. A little of the time
17. Some of the time
18. Most of the time
19. All of the time
20. How much pain or discomfort have you had in and around your eyes (for example, burning, itching, or aching)? Would you say it is:
21. None
22. Mild
23. Moderate
24. Severe
25. Very severe

PART 2 - DIFFICULTY WITH ACTIVITIES

The next questions are about how much difficulty, if any, you have doing certain activities wearing your glasses if you use them for that activity.

1. How much difficulty do you have reading ordinary print in newspapers? Would you say you have:
2. No difficulty at all
3. A little difficulty
4. Moderate difficulty
5. Extreme difficulty
6. Stopped doing this because of your eyesight
7. Stopped doing this for other reasons or not interested in
8. How much difficulty do you have doing work or hobbies that require you to see well up close, such as cooking, sewing, fixing things around the house, or using hand tools? Would you say:
9. No difficulty at all
10. A little difficulty
11. Moderate difficulty
12. Extreme difficulty
13. Stopped doing this because of your eyesight
14. Stopped doing this for other reasons/ not interested in doing this
15. Because of your eyesight, how much difficulty do you have finding something on a crowded shelf?
16. No difficulty at all
17. A little difficulty
18. Moderate difficulty
19. Extreme difficulty
20. Stopped doing this because of your eyesight
21. Stopped doing this for other reasons/ not interested in doing this
22. Because of your eyesight, how much difficulty do you have recognizing people you know from across a room?

1. No difficulty at all

2. A little difficulty

3. Moderate difficulty

4. Extreme difficulty

5. Stopped doing this because of your eyesight

6. Stopped doing this for other reasons/ not interested in doing this

1. Because of your eyesight, how much difficulty do you have going down steps, stairs, or curbs in dim light or at night?
2. No difficulty at all
3. A little difficulty
4. Moderate difficulty
5. Extreme difficulty
6. Stopped doing this because of your eyesight
7. Stopped doing this for other reasons/ not interested in doing this
8. Because of your eyesight, how much difficulty do you have noticing objects off to the side while you are walking along?
9. No difficulty at all
10. A little difficulty
11. Moderate difficulty
12. Extreme difficulty
13. Stopped doing this because of your eyesight
14. Stopped doing this for other reasons/ not interested in doing this
15. Because of your eyesight, how much difficulty do you have seeing how people react to things you say?
16. No difficulty at all
17. A little difficulty
18. Moderate difficulty
19. Extreme difficulty
20. Stopped doing this because of your eyesight
21. Stopped doing this for other reasons/ not interested in doing this
22. Because of your eyesight, how much difficulty do you have picking out and matching your own clothes?
23. No difficulty at all
24. A little difficulty
25. Moderate difficulty
26. Extreme difficulty
27. Stopped doing this because of your eyesight
28. Stopped doing this for other reasons/ not interested in doing this
29. Because of your eyesight, how much difficulty do you have visiting with people in their homes, at parties, or in restaurants?
30. No difficulty at all
31. A little difficulty
32. Moderate difficulty
33. Extreme difficulty
34. Stopped doing this because of your eyesight
35. Stopped doing this for other reasons/ not interested in doing this
36. Because of your eyesight, how much difficulty do you have going out to see movies, plays, or sports events?
37. No difficulty at all
38. A little difficulty
39. Moderate difficulty
40. Extreme difficulty
41. Stopped doing this because of your eyesight
42. Stopped doing this for other reasons/ not interested in doing this
43. Are you currently driving, at least once in a while?
44. Yes----- *Skip To Q 15c*
45. No

15a. IF NO: Have you never driven a car or have you given up driving?

1. Never drove ----- *Skip To Part 3, Q 17*
2. Gave up

15b. IF YOU GAVE UP DRIVING: Was that mainly because of your eyesight, mainly for some other reason, or because of both your eyesight and other reasons?

1. Mainly eyesight................................ *Skip To Part 3, Q 17*
2. Mainly other reasons....................... *Skip To Part 3, Q 17*
3. Both eyesight and other reasons ... *Skip To Part 3, Q 17*

15c. IF CURRENTLY DRIVING: How much difficulty do you have driving during the daytime in familiar places? Would you say you have:

1. No difficulty at all
2. A little difficulty
3. Moderate difficulty
4. Extreme difficulty
5. How much difficulty do you have driving at night? Would you say you have:
6. No difficulty at all
7. A little difficulty
8. Moderate difficulty
9. Extreme difficulty
10. Have you stopped doing this because of your eyesight
11. Have you stopped doing this for other reasons/ are you not interested in doing this

16A. How much difficulty do you have driving in difficult conditions, such as in bad weather, during rush hour, on the freeway, or in city traffic? Would you say you have?

1. No difficulty at all
2. A little difficulty
3. Moderate difficulty
4. Extreme difficulty
5. Have you stopped doing this because of your eyesight
6. Have you stopped doing this for other reasons/ are you not interested in doing this

PART 3: RESPONSES TO VISION PROBLEMS

The next questions are about how things you do may be affected by your vision. For each one, please choose the number to indicate whether for you the statement is true for you all, most, some, a little, or none of the time.

| CATEGORIES | All of the time | Most of  the time | A little  of the  time | Some  of the  time | None of  the time |
| --- | --- | --- | --- | --- | --- |
| 17. Do you accomplish less than you would like because of your vision? | 1 | 2 | 3 | 4 | 5 |
| 18. Are you limited in how long you can work or do other activities because of your vision? | 1 | 2 | 3 | 4 | 5 |
| 19. How much does pain or discomfort in or around your eyes, for example, burning, itching, or aching, keep you from doing what you’d like to be doing? | 1 | 2 | 3 | 4 | 5 |

PART 4: WELL-BEING/DISTRESS and DEPENDENCY

For each of the following statements, please choose the number to indicate whether for you the statement is definitely true, mostly true, mostly false, or definitely false for you or you are not sure.

|  | Definitely True | Mostly True | Not Sure | Mostly False | Definitely  False |
| --- | --- | --- | --- | --- | --- |
| 20. I stay home most of the time because of my eyesight | *1* | *2* | *3* | *4* | *5* |
| 21. I feel frustrated a lot of the time because of my eyesight | *1* | *2* | *3* | *4* | *5* |
| 22. I have much less control over what I do, because of my eyesight. | *1* | *2* | *3* | *4* | *5* |
| 23. Because of my eyesight, I have to rely too much on what other people tell me | *1* | *2* | *3* | *4* | *5* |
| 24. I need a lot of help from others because of my eyesight | *1* | *2* | *3* | *4* | *5* |
| 25. I worry about doing things that will embarrass myself or others, because of my eyesight | *1* | *2* | *3* | *4* | *5* |

Annex 5 Amharic version of questionnaire

የአማርኛመጠይቅቅጽ

መለያቁጥር -----------------

ጤና ይስጥልኝ እኔ ………………………………………እባላለሁ፡፡ የመጣሁት ከጎንደር ዩኒቨርስቲ ነዉ፡፡ ከእይታ መቀነስ እና አይነ-ስውርነት ጋር የሚኖሩ ሰዎች የሚገጥማቸውን የህይወት አኗኗር የጥራት ደረጃ እና ተያያዥ ምክንያቶች በሚደርገዉ ጥናት ና ምርምር አባል ነኝ፡፡ በጎንደር ዩኒቨርስቲ ሆስፒታል የአይን ህክምና ክፍል የመጡ ከእይታ መቀነስ እና አይነ-ስውርነት ጋር የሚኖሩ ሰዎች የሚገጥማቸውን የህይወት አኗኗር የጥራት ደረጃ እና ተያያዥ ምክንያቶች ዙሪያ የዳሰሳ ጥናት እያደረግኩ ነዉ፡፡ለሁሉም ጥያቄዎች የሚሰጡን ትክክለኛ መልስ ለማጠናዉ ጥናት በጣም ጠቃሚ ነዉ፡፡የርስዎ መረጃ ምስጢሩ የተጠበቀ ነዉ፡፡ በጥናቱ ላይ አሁንም ሆነ መሀል ላይ መሳተፍ ባይፈልጉ መብትዎ ነዉ፡፡ ነገር ግን ጥናቱ ከሚሰጠዉ ጥቅም አንፃር እንዲሳተፉ እንመክራለን፡፡መጠይቁን ለማጠናቀቅ 20 ደቂቃ ይወስዳል፡፡

አመስግናለሁ፤ከዚህ በመቀጠል ለመሳተፍ ፈቃደኝነትዎን የማረጋገጫ ጽሁፍ አነብሎታለሁ፡፡

ለመቀጠል ይስማማሉ?

ከተስማሙ አመሰግናለሁ፡፡ መጠይቁ ይቀጥላል

ካልተስማሙም አመስግነህ ወደ ቀጣዩ ተሳታፊ ሂድ

ማንኛውምሊያነሱየሚፈልጉትጥያቄካለዎትተመራማሪዎቹንበሚቀጥለውአድራሻማነጋገርይችላሉ፡፡

ስም፡ቤተልሄም ተመስገን፤ ደስታዬ ሽፈራው፤ ደረጀ ሀይሉ

ስ. ቁ፡0985066685, 0918032216,0910046930

መረጃ ሰብሳቢ

ስም………………………………………………………….. ፊርማ………………………………. ቀን………………………..

ያረጋገጠዉ ተቆጣጣሪ

ስም……………………………………………………………. ፊርማ………………………….. ቀን…………………………

**ክፍል 1: የምርምሩተሳታፊዎችንስነህዝባዊ እናማህበራዊሁኔታየሚያስስመጠይቅ**

| ተ.ቁ | ጥያቄ | መልስ |
| --- | --- | --- |
| 1 | ዕድሜ | _________ ዓመት |
| 2 | ፆታ | 1. ወንድ 2. ሴት |
| 3 | አድራሻ | 1. ከተማ 2. ገጠር |
| 4 | ብሄር | 1. አማራ 2. ኦሮሞ  3. ትግሬ 4. ሌላ( ይጠቀስ)--------- |
| 5 | የጋብቻ ሁኔታ | 1. ያላገባ 2. ያገባ  3. የፈታ 4. የትዳር አጋሩ የሞተበት |
| 6 | ሃይማኖት | 1. ክርስትያን 2. ሙስሊም |
| 7 | የትምህርት ደረጃ | 1. ማንበብ እና መፃፍ የማይችል  2. ማንበብ እና መፃፍ የሚችል  3. 1 – 8 ኛክፍል  4. 9 – 12 ኛክፍል  5. ኮሌጅ/ዩኒቨርስቲ |
| 8 | ስራ | 1. ተማሪ 2. የቤት እመቤት 3. ነጋዴ 4. የቀን ሰራተኛ 5. ገበሬ 6. የመንግስት ሰራተኛ 7. ጡረተኛ 8. ሌላ--------- |
| 9 | ወርሃዊ ገቢ | _____________የኢትዮጲያ ብር |

**ክፍል 3**፡ከእይታ መቀነስ ጋር ተያይዞየህይወት አኗኗር የጥራት ደረጃን በተመለከተ

ክፍል 1፡ አጠቃላይ ጤናን እና እይታን በተመለከተ

1. አጠቃላይ ጤናዎትን እንዴት ይገልፁታል

1. እጅግ በጣም ጥሩ

1. በጣም ጥሩ
2. ጥሩ
3. መጠነኛ
4. ዝቅተኛ
5. በአሁኑ ሰአት በሁለት አይንዎት ያለዎትን እይታ እንዴት ይገልፁታል(መነፀር የሚጠቀሙ ከሆነ በመነፀር ያለዎትን እይታ)
6. እጅግ በጣም ጥሩ
7. በጣም ጥሩ
8. ጥሩ
9. መካከለኛ
10. ዝቅተኛ
11. ምንም ነገር ማየት አልችልም
12. ምን ያህል ጊዜዎን ስለእይታዎ መቀነስ በመጨነቅ ያጠፋሉ?
13. ምንም ጊዜ አልጨነቅም
14. ለትንሽ ጊዜ እጨነቃለሁ
15. አልፎ አልፎ እጨነቃለሁ
16. አብዛኛውን ጊዜ እጨነቃለሁ
17. ሁሌምእጨነቃለሁ
18. ምን ያህል የአይን ህመም ወይም ውዝዋዜ አለብዎት ለምሳሌ እንደማቃጠል፣ማሳከክ ወይም መወዝወዝ?
19. ምንም ችግር የለብኝም
20. ትንሽ ችግርአለብኝ
21. መካከለኛ ችግር አለብኝ
22. ከፍተኛ ችግር አለብኝ
23. በጣም ከፍተኛ ችግር አለብኝ

ክፍል 2፡ የእለት ከእለት እንቅስቃሴዎችን በተመለከተ

1. ምን ያህል የማንበብ ችግር አለብዎት?
2. ምንምአልቸገርም
3. ዝቅተኛ ችግር
4. መካከለኛ ችግር
5. ከፍተኛ ችግር
6. በእይታ መቀነስ ምክንያት ማንበብ አቁሜአለሁ
7. በሌላ ችግር ማንበብ አቁሜአለሁ/ በፊትም አላነብም ነበር
8. ምን ያህል በጥራት ማየት የሚያስፈልጋቸውን ስራዎች ለምሳሌ ምግብ ማብሰል፣ ልብስ መስፋት ወይም ቤት ማስተካከል ይቸገራሉ?
9. ምንምአልቸገርም
10. ዝቅተኛ ችግር
11. መካከለኛ ችግር
12. ከፍተኛ ችግር
13. በእይታ መቀነስ ምክንያት የቤት ስራወችን መስራትአቁሜአለሁ
14. በሌላ ችግር የቤት ስራወችን መስራትአቁሜአለሁ/ በፊትም የቤት ስራወችን አልሰራምነበር
15. እይታዎ መቀነስ ምክንያት ምን ያህል እቃዎችን ለማግኘት ይቸገራሉ?
16. ምንምአልቸገርም
17. ዝቅተኛ ችግር
18. መካከለኛ ችግር
19. ከፍተኛ ችግር
20. በእይታ መቀነስ ምክንያት እቃዎችን ማግኘት አቁሜአለሁ
21. በሌላ ችግር እቃዎችን ማግኘት አቁሜአለሁ/ በፊትም እቃዎችን አልፈልግም ነበር
22. በእይታ መቀነስ ምክንያት ምን ያህል የሚያውቁአቸውን ሰወች ከርቀት ለመለየት ይቸገራሉ?

1. ምንም አልቸገርም

2. ዝቅተኛ ችግር

3. መካከለኛ ችግር

4. ከፍተኛ ችግር

5. በእይታ መቀነስ ምክንያት የማውቃቸውን ሰወች ከርቀት መለየት አቁሜአለሁ

6. በሌላ ችግር የማውቃቸውን ሰወች ከርቀት መለየት አቁሜአለሁ/ በፊትም የማውቃቸውን ሰወች ከርቀት መለየት አልችልም ነበር

1. በእይታዎ መቀነስ ምክንያት ምን ያህል በምሽት ደረጃ ለመውጣት/ለመውረድ ይቸገራሉ?
2. ምንምአልቸገርም
3. ዝቅተኛ ችግር
4. መካከለኛ ችግር
5. ከፍተኛ ችግር
6. በእይታ መቀነስ ምክንያት በምሽት ደረጃ መውጣት/መውረድ አቁሜአለሁ
7. በሌላ ችግር በምሽት ደረጃ መውጣት/መውረድ አቁሜአለሁ/ በፊትም በምሽት ደረጃ አልወጣም/አልወርድም ነበር
8. በእይታዎ መቀነስ ምክንያት ምን ያህል እየተንቀሳቀሱ በጎን ያሉ ነገሮችን ለማየት ይቸገራሉ?
9. ምንምአልቸገርም
10. ዝቅተኛ ችግር
11. መካከለኛ ችግር
12. ከፍተኛ ችግር
13. በእይታ መቀነስ ምክንያት በጎን ያሉ ነገሮችን ማየት አቁሜአለሁ
14. በሌላ ችግር በጎን ያሉ ነገሮችን ማየት አቁሜአለሁ/ በፊትም በጎን ያሉ ነገሮችን አላይም ነበር
15. በእይታዎ መቀነስ ምክንያት ምን ያህል ለተናገሩት ነገር የሰወችን መልስ ለመረዳት ይቸገራሉ?
16. ምንምአልቸገርም
17. ዝቅተኛ ችግር
18. መካከለኛ ችግር
19. ከፍተኛ ችግር
20. በእይታ መቀነስ ምክንያት የሰወችን መልስ መረዳት አቁሜአለሁ
21. በሌላ ችግር የሰወችን መልስ መረዳት አቁሜአለሁ/ በፊትም የሰወችን መልስ አልረዳም ነበር
22. በእይታዎ መቀነስ ምክንያት ምን ያህል የልብስዎትን ቀለም ለመለየት ይቸገራሉ?
23. ምንምአልቸገርም
24. ዝቅተኛ ችግር
25. መካከለኛ ችግር
26. ከፍተኛ ችግር
27. በእይታ መቀነስ ምክንያት የልብሴን ቀለም መለየት አቁሜአለሁ
28. በሌላ ችግር የልብሴን ቀለም መለየት አቁሜአለሁ/ በፊትም የልብሴን ቀለም አልለይም ነበር
29. በእይታዎ መቀነስ ምክንያት ምን ያህል ሰወችን ቤታቸው ወይም ሌላ ቦታ ሄደው መጠየቅ ይቸገራሉ?
30. ምንምአልቸገርም
31. ዝቅተኛ ችግር
32. መካከለኛ ችግር
33. ከፍተኛ ችግር
34. በእይታ መቀነስ ምክንያት ሰወችን ቤታቸው ወይም ሌላ ቦታ ሄጄ መጠየቅአቁሜአለሁ
35. በሌላ ችግር ሰወችን ቤታቸው ወይም ሌላ ቦታ ሄጄ መጠየቅ አቁሜአለሁ/ በፊትም ሰወችን ቤታቸው ወይም ሌላ ቦታ ሄጄ አልጠይቅምነበር
36. በእይታዎ መቀነስ ምክንያት ምን ያህል ከቤት ውጪ ለመዝናናት ወይም ስፖርታዊ ክንዋኔዎችን ለማድረግ ይቸገራሉ?
37. ምንምአልቸገርም
38. ዝቅተኛ ችግር
39. መካከለኛ ችግር
40. ከፍተኛ ችግር
41. በእይታ መቀነስ ምክንያት ከቤት ውጪ መዝናናት ወይም ስፖርታዊ ክንዋኔዎችን ማድረግ አቁሜአለሁ
42. በሌላ ችግር ከቤት ውጪ መዝናናት ወይም ስፖርታዊ ክንዋኔዎችን ማድረግ አቁሜአለሁ/ በፊትም ከቤት ውጪ አልዝናናም ወይም ስፖርታዊ ክንዋኔዎችን አላደርግም ነበር
43. አሁን ወይም ከዚህ በፊት መኪና አሽከርክረው ያውቃሉ?
44. አውቃለሁ----- ወደ ጥያቄ 15ሐ ሂድ 2. አላውቅም

15ሀ. የተራ ቁጥር 15 መልስ አላውቅም ከሆነ፣ ከዚህ በፊት መኪና ነድተው አያውቁም ወይስ መንዳት አቁመው ነው?

1. ነድቼ አላውቅም ------------ ወደ ጥያቄ *17ሂድ 2.* መንዳት አቁሜአለሁ

15ለ. የተራ ቁጥር 15ሀ መልስ አቁሜአለሁ ከሆነ፣ ለምን?

1. በእይታ መቀነስ ምክንያት--------- ወደ ጥያቄ *17ሂድ*
2. በሌላ ችግር ምክንያት-------------ወደ ጥያቄ *17ሂድ*
3. በሁለቱም ምክንያት -------------- ወደ ጥያቄ *17ሂድ*

15ሐ. የተራ ቁጥር 15 መልስ አውቃለሁ ከሆነ፣ በቀን በሚያውቁት አካባቢ መኪና ሲነዱ ምን ያህል ይቸገራሉ?

1. ምንምአልቸገርም
2. ዝቅተኛ ችግር
3. መካከለኛ ችግር
4. ከፍተኛ ችግር
5. በምሽት መኪና ሲነዱ ምን ያህል ይቸገራሉ?
6. ምንምአልቸገርም
7. ዝቅተኛ ችግር
8. መካከለኛ ችግር
9. ከፍተኛ ችግር
10. በእይታ መቀነስ ምክንያት በምሽት መኪና መንዳት አቁሜአለሁ
11. በሌላ ችግር በምሽት መኪና መንዳት አቁሜአለሁ/ በፊትም በምሽት መኪና አልነዳም ነበር

16ሀ. በአስቸጋሪ ሁኔታዎች ውስጥ ለምሳሌ በአስቸጋሪ የአየር ንብረት፣በቸኮሉ ጊዜ ወይም በተጨናነቀ መንገድ መኪና ሲያሽከረክሩ ምን ያህል ይቸገራሉ?

1. ምንም አልቸገርም

2. ዝቅተኛችግር

3. መካከለኛችግር

4. ከፍተኛችግር

5. በእይታ መቀነስ ምክንያት በአስቸጋሪ ሁኔታዎች ውስጥ መኪና መንዳት አቁሜአለሁ

6. በሌላ ችግር በአስቸጋሪ ሁኔታዎች ውስጥ መኪና መንዳት አቁሜአለሁ/ በፊትም በአስቸጋሪ ሁኔታዎች ውስጥ መኪና አልነዳም ነበር

ክፍል 3፡ የእይታ ችግር ተፅእኖን በተመለከተ

| ጥያቄ | ሁልጊዜ | በአብዛኛው | አንዳንዴ | አልፎ አልፎ | ምንም ጊዜ |
| --- | --- | --- | --- | --- | --- |
| 17.በእይታ መቀነስ ምክንያት መስራት የፈለጉትን ነገር ካሰቡት ወይም ከፈለጉት በታች ይፈፅማሉ? | 1 | 2 | 3 | 4 | 5 |
| 18. በእይታ መቀነስ ምክንያት የስራዎን ወይም የሌላ ክንውኖችን ሰአት ይቀንሳሉ? | 1 | 2 | 3 | 4 | 5 |
| 19. የአይንዎት ህመም ወይም ስቃይ ለምሳሌ ማቃጠሉ፣ማሳከኩ ወይም መወዝወዙ የፈለጉትን ስራ እንዳይሰሩ አድርጎዎታል? | 1 | 2 | 3 | 4 | 5 |

ከፍል 4፡ ደህንነት፣መጨናነቅ እና ጥገኝነትን በተመለከተ

| *ጥያቄ* | በእርግጠኝነት እውነት | በአብዛኛው እውነት | እርግጠኛ አይደለሁም | በአብዛኛው ሀሰት | በእርግጠኝነት ሀሰት |
| --- | --- | --- | --- | --- | --- |
| 20. በእይታ መቀነስ ምክንያት ብዙ ጊዜ ቤት ውስጥ ይውላሉ፡፡ | *1* | *2* | *3* | *4* | *5* |
| 21. በእይታ መቀነስ ምክንያት ብዙ ጊዜ መናደድ፣ ተስፋ መቁረጥ ስሜት ይሰማዎታል፡፡ | *1* | *2* | *3* | *4* | *5* |
| 22. በእይታ መቀነስ ምክንያት ስራዎትን መቆጣጠር እይችሉም፡፡ | *1* | *2* | *3* | *4* | *5* |
| 23. በእይታ መቀነስ ምክንያት ሌሎች ሰወች በሚነግሩዎት ነገር ላይ የተማመናሉ፡፡ | *1* | *2* | *3* | *4* | *5* |
| 24. በእይታ መቀነስ ምክንያት ከሌሎች ሰወች ብዙ እርዳታ ይፈልጋሉ፡፡ | *1* | *2* | *3* | *4* | *5* |
| 25. በእይታ መቀነስ ምክንያት እርስዎንም ሆነ ሌላ ሰወችን የሚያሳፍር ስራ እንዳይሰሩይጨነቃሉ፡፡ | *1* | *2* | *3* | *4* | *5* |
